# Pigs in southern Italy are exposed to three ruminant pathogens: an analysis of seroprevalence and risk factors analysis study

**DOI:** 10.1186/s12917-024-04037-4

**Published:** 2024-05-08

**Authors:** Gianmarco Ferrara, Ugo Pagnini, Elvira Improda, Giuseppe Iovane, Serena Montagnaro

**Affiliations:** Department of Veterinary Medicine and Animal Productions, University of Naples Federico II, Via Federico Delpino n.1, Naples, 80137 Italy

**Keywords:** Schmallenberg virus, Coxiella, BVD, Seroprevalence, Interspecies transmission, Pigs, Italy

## Abstract

**Background:**

Pigs are susceptible to several ruminant pathogens, including *Coxiella burnetti*, Schmallenberg virus (SBV) and bovine viral diarrhea virus (BVDV). These pathogens have already been described in the pig population, although the dynamics of the infection and the impact on pig farms are currently unclear. The aim of this work was to evaluate the presence of these infections in the pig population of the Campania region, southern Italy, and to evaluate the risk factors associated with a greater risk of exposure.

**Results:**

A total of 414 serum samples belonging to 32 herds were tested for the presence of antibodies against SBV, *Coxiella*, and BVD using commercial multispecies ELISA kits. SBV (5.3%) was the most prevalent pathogen, followed by *Coxiella* (4.1%) and BVD (3%). The risk factors included in the study (age, sex, province, farming system, ruminant density and major ruminant species) had no influence on the probability of being exposed to BVD and *Coxiella*, except for the location, in fact more pigs seropositive to *Coxiella* were found in the province of Caserta. However, the univariate analysis highlighted the influence of age, location, and sex on exposure to SBV. The subsequent multivariate analysis statistically confirmed the importance of these factors. The presence of neutralizing antibodies for SBV and BVDV, or antibodies directed towards a specific phase of infection for *Coxiella* was further confirmed with virus-neutralization assays and phase-specific ELISAs in a large proportion of positive samples. The presence of high neutralizing antibody titers (especially for SBV) could indicate recent exposures. Twelve of the 17 positive samples tested positive for antibodies against Coxiella phase I or II antigens, indicating the presence of both acute and chronic infections (one animal tested positive for both phases antibodies).

**Conclusions:**

Our study indicates a non-negligible exposure of pigs from southern Italy to the above pathogens. Further studies are necessary to fully understand the dynamics of these infections in pigs, the impact on productivity, and the public health consequences in the case of *Coxiella*.

**Supplementary Information:**

The online version contains supplementary material available at 10.1186/s12917-024-04037-4.

## Background

Pigs (*Sus scrofa*) are susceptible to numerous infections that affect ruminants. Although these infections cause symptoms and economic losses in ruminants (considered the main hosts), they cause asymptomatic infections in pigs in most cases [1]. Swine, both domestic and wild, can become accidentally infected due to direct or indirect contact with infected ruminants or through active or passive vectors. For some of these pathogens, intraspecies transmission is still the subject of debate in the scientific community [1]. Examples of typical ruminant infections to which pigs are susceptible are Q fever, Schmallenberg virus (SBV), and bovine viral diarrhea (BVD) [2–4].

Q fever, caused by the Gram-negative bacterium *Coxiella burnetii*, is responsible for reproductive disorders (placenta retention, abortion, and metritis) and economic losses in domestic ruminants [5]. Coxiella has a broad host range and can potentially infect all species of mammals (including swine). Knowledge about the susceptibility of pigs to Q fever, as well as information about its clinical and economic impact, is very scarce [2, 6]. Information about this infection in pigs is currently limited to reports documenting molecular detection inorgans and body fluids as well as the presence of antibodies, while it remains undefined if *C. burnetii* is an abortigenic agent in pigs. Abortion in pigs is possible considering comparative aspects [6, 7]. Since it is still unclear whether pigs can transmit the infection and pose a public health issue, investigations aimed at identifying the level of exposure of pig populations are advisable.

SBV is an enveloped arbovirus belonging to the Bunyaviridae family and is transmitted through the bite of *Culicoides* among ruminants [8]. This virus caused a severe outbreak throughout Europe in 2012, including reproductive disorders (abortion and teratogenic lesions) [9]. The susceptibility of non-ruminant mammals has been studied and evaluated over the years, even with experimental infections [3]. Several studies have shown that, while pigs produce antibodies in response to contact with SBV documented in both domestic and wild pigs, they do not develop clinical symptoms or eliminate the virus, and, given the lack of viremia following experimental inoculation, they should probably be considered dead-end hosts [3]. Moreover, during experimental infections, several SBV target organs, such as the spleen, lymph nodes, brain, etc. resulted negative to molecular assay [3]. The ability to infect porcine cells is also preserved in vitro; in fact, SBV is able to grow on porcine-derived cells such as porcine kidney cells (SK-6) [10].

Bovine viral diarrhea virus (BVDV) is a pestivirus responsible for enteric disease, immunosuppression, and early abortion in cattle [11]. BVD is considered one of the diseases with the highest economic impact on the cattle industry. Due to its genomic similarities with classical swine fever virus (CSFV; both are pestiviruses), the virus can also infect pigs [12]. BVD has been described in numerous reports in both pigs and wild boars, and scientific evidence has demonstrated seroconversion and viral shedding with various body fluids after experimental infection [4, 13–15]. Further studies have highlighted the absence of persistent and transplacental infections in pigs [16–18].

The aim of this work was to evaluate the exposure of domestic pigs in the Campania region, southern Italy, to three different ruminant pathogens (SBV, BVDV and Coxiella) considered endemic in the study area, evaluating any risk factors involved in the spread of these pathogens in the pig population.

## Methods

### Study area and sampling

This study was conducted in the Campania region (41.1099° N, 14.8475° E), located in southern Italy. This area has a Mediterranean climate, which allows the epidemiologic cycle of viruses transmitted by vectors (*Culex*, *Culicoides*, etc.) [19, 20]. The largest type of farming involves ruminants (mainly buffalo), while pig farming counted 75,000 animals at the time of sampling (2% of the national total; Banca Dati Nazionale dell’ Anagrafe Zootecnica, https://www.vetinfo.it/j6statistiche/, accessed November 15, 2022). We selected an expected prevalence of 0.5 (i.e., 50%), an absolute precision of 5%, and a confidence interval (CI) of 95% due to a lack of similar surveys in the same area. The sample size was determined using Thrusfield’s formula in the Epi Info software:

n = Z 2 × P(1 − P)/ d 2 where: Z = 1.96 for a confidence level of 95%, P = expected prevalence, d = 0.05 accepted error and n = sample size.

A total of 414 blood samples were randomly collected from 32 farms (randomly selected from those listed in the study area) [21]. Sampling coincided with blood collection by state veterinarians for the national pseudorabies eradication campaign and was part of another investigation (ethical approval was not required).

### Enzyme-Linked Immunosorbent Assays

Three commercial and multispecies ELISAs were used to assess the detection of antibodies against BVDV, SBV, and Coxiella: ID Screen® BVD p80 Antibody Competition, ID Screen® Schmallenberg virus Competition Multi-species, ID Screen® Q Fever Indirect Multi-species (Innovative diagnostics, ID.vet). All tests were carried out following the manufacturer’s instructions. Although validated in ruminants, these tests have been used in porcine species in previous studies due to their nature (ID Screen® BVD p80 Antibody Competition and ID Screen® Schmallenberg virus Competition Multi-species are competitive ELISAs, while ID Screen® Q Fever Indirect Multi-species is an indirect ELISA that uses a multispecies conjugated secondary antibody). A spectrophotometer (Thermo Fisher Scientific) was used to detect optical density, which was subsequently used to calculate the cut-off value that differentiated a positive from a negative sample.

### Statistical analysis

The number of positive pigs was divided by the total number of pigs investigated to determine the prevalence at the animal level. Univariate analysis (chi-square test) was conducted using the ELISA results for each pathogen (as dependent variables) and the information about potential risk factors (as independent variables). Pigs were categorized according to location (Avellino, Benevento, Caserta, Napoli, and Salerno), sex (male or female), and farm system (intensive or extensive based on the presence of pasture). Furthermore, pigs were considered adult (sows or boars) or young (growers or finishers). The total number of each ruminant species was calculated via the national database (selecting the main species bred in each district), which, divided by the surface area of the district, resulted in ruminant density per square kilometer. P-values less than 0.05 were considered significant (MedCalc Statistical Software, Ostend, Belgium, version 16.4.3). Using the forward elimination approach, all significant variables were evaluated for the multivariate logistic regression. The degree of correlation between independent variables and SBV seropositivity (the only pathogen for which multiple risks associated with higher exposures have been identified) was assessed using odds ratios (OR) and 95% confidence intervals. The Akaike Information Criterion (AIC) was used to evaluate fit models, and those that best fit the data were chosen. To test for collinearity, the Variance inflation factor (VIF) was employed.

### Serological confirmation

The detection of antibodies against BVDV and SBV was further confirmed by subjecting the same samples to a virus neutralization assay. In both cases, protocols described in the literature were used, cultivating MDBK and BHK-21 cells in 96-well plates, susceptible to BVDV and SBV, respectively [19, 22, 23]. Serial serum dilutions from 1:2 to 1:256 (inactivated at 56 °C for 30 min) were mixed with 100 TCID50 of BVDV strain NADL (ATCC) and 500 TCID50 of SBV strain BH80/11–4 (gently provided by “Friedrich-Loefer-Institut”, Germany and “Istituto Zooproflattico Sperimentale dell’Abruzzo e del Molise G. Caporale”, Italy). The serum and virus were pre-incubated overnight at 37 °C before being introduced to the cells and incubated for four days. The titer was calculated as the highest dilution able to prevent cytopathic effects in triplicate wells.

Two phase-specific ELISAs (Coxiella burnetii phase 1 and 2, Euroimmun, Germany) were used according to the manufacturer’s instructions to confirm the specificity of the anti-Coxiella antibodies. This method was feasible to determine whether reactivity was directed towards phase I (chronic form) or entirely towards phase II (acute form) [24, 25].

## Results

A total of 414 pigs were tested with multispecies ELISAs, obtaining a seroprevalence of 5.3% for SBV (which resulted in the most widespread pathogen), 4.1% for Coxiella, and 2.9% for BVDV (Tables 1, 2 and 3). A single co-exposure was observed in a pig exhibiting antibodies against BVDV and SBV. Herd prevalence was higher for all three pathogens investigated (BVD 18.75%, Coxiella 34.4%, and SBV 31.2%). The univariate analysis of the risk factors revealed no significant results for BVD, with only the “sex” variable (female) being approximately associated with higher seroprevalences (a chi-square test p-value of 0.05 was obtained) (Table 1). However, in the case of Coxiella, greater exposure was observed in pigs raised in the province of Caserta (*p* = 0.03) (Table 2). The number of ruminants and the species of ruminants mainly reared in the district did not influence the seroprevalence of any of the three infections. The univariate analysis of risk factors associated with SBV seropositivity provided further interesting results (Table 3). In particular, the provinces of Caserta, Naples, and Salerno (*p* < 0.001) had higher seroprevalences than the others. Furthermore, sex and age were also found to be risk factors significantly associated with greater risks of exposure, as females (*p* = 0.001) and mature animals (*p* < 0.001) had a greater risk of being seropositive. Subsequent multivariate analysis (logistic regression) confirmed the significantly higher exposure for female (*p* = 0.003) and mature animals (*p* = 0.002) raised in the province of Salerno (*p* = 0.002) (Table 4). Risk factors concerning the density and the main ruminant species raised in the study area were not correlated with higher prevalences.

**Table 1:** Univariate analysis (chi-square) of individual risk factors (province, sex, age, and farm system) for BVDV seropositivity

	BVD					
Factor	n	Positive	%	95%CI	χ^2^	**p**
Total	414	12	3	1.3–4.5		
Province						
Avellino	141	7	5	1.4–8.6		
Benevento	95	1	1	0.0–3.1		
Salerno	48	2	4.2	0.0–9.8	5.9	0.23
Caserta	63	2	3.2	0.0–7.5		
Napoli	67	0	0	0.0–0.0		
Sex						
Male	250	4	1.6	0.0–3.2		
					3.8	0.05
Female	164	8	4.9	1.6–8.2		
Age						
Growner/Finisher	291	10	3.4	1.8–7.3		
					1	0.31
Adult	123	2	1.6	0.0–3.9		
Farm system						
Intensive	337	12	3.6	1.6–5.5		
					2.8	0.09
Extensive	77	0	0	0.0–0.0		
Ruminants density (head/km^2^)						
≤ 50	210	6	2.8	0.6–5.1		
					0.0	0.95
> 50	204	6	2.9	0.6–5.3		
Major ruminant species						
Bovine	163	2	1.2	0–2.9		
Buffalo	163	7	4.3	1.2–7.4	2.8	0.24
Small ruminants	88	3	3.4	0–7.2		

**Table 2:** Univariate analysis (chi-square) of individual risk factors (province, sex, age, and farm system) for *Coxiella burnetii* seropositivity

	Coxiella					
Factor	n	Positive	%	95%CI	χ^2^	**p**
Total	414	17	4.1	2.2–6.0		
Province						
Avellino	141	5	3.5	0.5–6.6		
Benevento	95	0	0	0–0		
Salerno	48	1	2.1	0.0–6.1	11.3	**0.03**
Caserta	63	6	9.5	2.3–16.8		
Napoli	67	5	7.5	1.2–13.8		
Sex						
Male	250	11	4.4	1.9–6.9		
					0.14	0.71
Female	164	6	3.7	0.8–6.5		
Age						
Growner/Finisher	291	11	3.8	1.6–6.0		
					0.26	0.26
Adult	123	6	4.9	1.1–8.7		
Farm system						
Intensive	337	16	4.7	2.5–7.0		
					1.9	0.17
Extensive	77	1	1.3	0.0–3.8		
Ruminants/km^2^						
≤ 50	210	11	5.2	2.2–8.2		
					1.4	0.23
> 50	204	6	2.9	0.6–5.3		
Major ruminant species						
Bovine	163	8	4.9	1.6–8.2		
Buffalo	163	7	4.3	1.2–7.4	1	0.59
Small ruminants	88	2	2.2	0–5.4		

**Table 3:** Univariate analysis (chi-square) of individual risk factors (province, sex, age, and farm system) for SBV seropositivity

	SBV					
Factor	n	Positive	%	95%CI	χ^2^	**p**
Total	414	22	5.3	3.2–7.5		
Province						
Avellino	141	2	1.4	0.0–3.4		
Benevento	95	2	2.1	0.0–5.0		
Salerno	48	7	14.6	4.6–24.6	20.3	**< 0.001**
Caserta	63	3	4.8	0.0–10.0		
Napoli	67	8	11.9	4.2–19.7		
Sex						
Male	250	6	2.4	0.5–4.3		
					10.6	**0.001**
Female	164	16	9.7	5.2–14.3		
Age						
Growner/Finisher	291	7	2.4	0.7–4.2		
					16.5	**< 0.001**
Adult	123	15	12.2	6.4–18.0		
Farm system						
Intensive	337	19	5.6	3.2–8.1		
					0.4	0.54
Extensive	77	3	3.9	0.0–8.2		
Ruminants/km^2^						
≤ 50	210	12	5.7	2.6–8.9		
					0.1	0.7
> 50	204	10	4.9	1.9–7.9		
Major ruminant species						
Bovine	163	9	5.5	2–9		
Buffalo	163	6	3.7	0.8–6.6	2.1	0.35
Small ruminants	88	7	7.9	2.3–13.6		

**Table 4:** Logistic regression model for the association of potential risk factors (*p* < 0.05) with SBV seropositivity

SBV				
Factor	Coefficient (β)	OR	95% CI	p-value
Age (Growner/Finisher)	-1.78	0.17	0.05–0.52	0.002
Province (Benevento)	0.08	1.1	0.13–8.7	0.94
Province (Caserta)	1.89	6.6	0.9–46.6	0.06
Province (Napoli)	1.2	3.2	0.6–19.3	0.16
Province (Salerno)	2.7	14.4	2.7–76.6	0.002
Sex (Male)	-1.62	0.2	0.07–0.58	0.003

Subsequently, the positive animals were tested with further serological assays (VTN and phase-specific ELISA). A large number of BVDV (11/12) and SBV (19/22) positive samples were verified by the corresponding VTN protocols. Some samples revealed very high antibody titers for SBV (six samples had a titer of 1:128 or more), suggesting a recent exposure. Antibody titers obtained with BVDV-positive samples were less high (only one sample had a titre of 1:128, all others were between 1:8 and 1:64). The results obtained in the phase-specific ELISAs confirmed the presence of anti-phase II antibodies in 12 animals and anti-phase I antibodies in only one pig (also positive in phase II) (Supplementary Data 1).

## Discussion

Pigs are susceptible to numerous ruminant infections, which they can contract in different ways, such as through proximity to herds of different species, exposure to vectors, or contact with wild animals [1, 26]. In this study, exposure to SBV, BVDV, and Coxiella was demonstrated in the pig population in the Campania region of southern Italy.

The highest seroprevalence was found for SBV, which can be transmitted to pigs through the blood meal of infected vectors. Recent studies have shown that SBV infection is widespread among ruminants in the same region (40.5%); consequently, it was not unexpected that pigs in this area were also exposed. Morevoer, the presence of numerous high antibody titers indicated recent exposure [20, 27]. During the outbreaks that occurred in Europe, the scientific community’s attention was concentrated more on wild boar, suspected of being a reservoir, than on pigs [28]. Several studies, in fact, have identified specific anti-SBV antibodies in populations of hunted wild boars. The prevalences would also seem to follow the typical temporal dynamics of the infection (linked to the activity of the vector). The percentages of positive animals obtained in other studies are between 0 and 3% (in Spain and Poland) [28–30]. During the first SBV epidemic in Europe, a study performed in Germany, one of the most affected countries, reported that 33% of wild boars tested positive in 2012, while only 11% tested positive the following year [31]. The absence of molecular positivity as well as experimental infection have indicated that the virus is not transmitted between pigs and that it is possible to consider the pig as a dead-end host (although porcine cells have been susceptible to SBV in vitro) that does not develop symptoms [3, 10]. Its role in this infection could therefore be that of a sentinel for interspecies transmission.

The analysis of risk factors has highlighted higher prevalences in certain provinces (Salerno, Caserta, and Naples) as well as in adult animals. The first aspect could be explained by the climatic characteristics of the above-mentioned provinces, which have a milder climate than the other locations belonging to internal areas. In fact, average temperature and the proximity to the coast favor the activity of the vector and, therefore, the spread of SBV and the possibility of it being transmitted to pigs [19, 20]. The second aspect (age), however, is explained by the fact that boars and sows have a greater chance of being seropositive as they experience multiple vector seasons. Further explanations for this result are weaker immune systems and more frequent secondary infections that affect older animals. Sex, type of farming system, ruminant density and the presence of other ruminants were not significant risk factors associated with BVDV, SBV and Coxiella exposure. These factors presumably do not play a primary role in influencing exposure to the pathogens mentioned above.

BVDV, in addition to being an important pathogen of ruminants, can affect pigs, being responsible for cross-reactions in serological tests to detect antibodies against classical swine fever [32]. Since Italy has been free of CSF for several years, further investigations were not required to distinguish between BVDV and CFSV antibodies. However, the test used is not able to differentiate antibodies against BVD from those against BDV (border disease virus), which shares the p-80 antigen used in the ELISA. In the absence of evidence of BD circulation in southern Italy, some cross-reactions may have influenced (and overestimated) BVD seroprevalence.

Furthermore, evidence of the identification of the pathogen in the secretions of infected pigs has been repeatedly reported (these can transmit the infection to other susceptible animals) [14]. The prevalence we observed (2.9%) is very similar to that described in other large-scale studies carried out in the Netherlands (2.5% in sows and 0.42% in finishing pigs) and Brazil (5.35% testing 1,705 sera by VNT) [32, 33]. Serological and molecular evidence has been described in numerous countries, including in wild boar. For example, specific antibodies are described in 1% of wild boars in the Czech Republic and in 5.4% of wild boars in Turkey [34, 35]. BVDV RNA was identified in 4/50 spleens and 3/49 lungs of wild boars hunted in Serbia and Brazil, respectively [36, 37]. Direct or indirect contact with infected wild boars, therefore, is a further chance of contagion for both pigs and ruminants.

Knowledge of coxiellosis in pigs is limited from a clinical and epidemiological point of view [6]. Reports of infection in domestic and wild swine populations both at serological and molecular levels are described in the literature and although no outbreaks related to pigs have been described to date, the zoonotic potential cannot be ruled out [2, 6]. In this case, the seroprevalences we obtained (4.1%) are very similar to those described in other studies using similar approaches, while the prevalence of ruminants is described as 11.7% in the study area. A large-scale study carried out in Korea in 2015 highlighted a seroprevalence of 6.8% in ELISA (5.2% of total samples were confirmed positive using immunofluorescence) and a molecular prevalence of 0.3% (testing 637 pigs) [7]. Similarly, evidence of Q fever, both serological and molecular, has been described in wild boars, providing evidence for the wildlife-livestock-human interface [38–41]. The univariate analysis revealed higher seroprevalences in the Caserta province, where the spread of Q fever in buffalo and cattle had previously been documented [42]. Not all ELISA-positive (12/17) pigs were confirmed in the phase-specific ELISA (several cross-reactions with *Bartonella* spp., *Chlamydia* spp., and *Rickettsiae* spp. should be considered), and in one animal it was possible to find antibodies against both phases of the pathogen (a finding similar to a chronic infection in ruminants) [25, 43]. A study recently conducted in Korea identified a seroprevalence of 14.6% in wild boars, identifying only environmental variables (mean annual temperature) instead of zootechnical ones as risk factors related to greater exposure [43].

Pigs and ruminants share numerous pathogens in addition to those described in this study, including viruses (such as foot-and-mouth disease, hepatitis E, West Nile virus etc.), bacteria (such as Brucella, Leptospira, Mycobacterium, etc.), and protozoa (such as Toxoplasma, etc.) [1, 44–47]. Evaluating the exchange of pathogens between the different species present in a territory is a veterinary prerogative, which leads to the concept of “one health” when humans are also involved in this cycle.

## Conclusions

Cross-species transmission of pathogens between domestic animals is a growing veterinary health concern. In this study, exposure to three abortigenic ruminant pathogens in the pig population in southern Italy was evaluated. Although obtained using a small sample of the pig population, our data highlights that pigs can inadvertently come into contact with these pathogens, even if further evidence (such as molecular detection) is needed to understand the dynamics of these cross-species transmissions, the consequences for animal health, and the risk to humans (for zoonotic infections).

### Electronic supplementary material

Below is the link to the electronic supplementary material.

Supplementary Material 1

## Data Availability

No datasets were generated or analysed during the current study.

## Abbreviations

SBV: Schmallenberg virus
BVDV: Bovine viral diarrhea virus
ELISA: Enzyme linked immunosorbent assay
SK-6: Swine kidney
CSFV: Classical swine fever virus
OR: Odds ratio
AIC: Akaike Information Criterion
VIF: Variance inflation factor
MDBK: Madin Darby Bovine Kidney
BHK-21: Baby hamster kidney
TCID50: Tissue Culture Infectious Dose
VNT: Virus neutralization
