# Supplementary material for: Pigs in southern Italy are exposed to three ruminant pathogens: an analysis of seroprevalence and risk factors analysis study
Source: BMC Vet Res. 2024 May 8;20:183. doi: 10.1186/s12917-024-04037-4 (PMC11077783; doi:10.1186/s12917-024-04037-4)
Supplement: Supplementary file 1 — Supplementary Material 1 [file 12917_2024_4037_MOESM1_ESM.docx]

| ID Sample | Positive | Titer (or ELISA optical density) | Province | Age | Sex | Type |
| --- | --- | --- | --- | --- | --- | --- |
| AV 45-1 | BVDV | 1:16 | Avellino | Finisher | Female | Intensive |
| AV 47-2 | BVDV | 1:32 | Avellino | Growner | Female | Intensive |
| AV 49-3 | BVDV | 1:16 | Avellino | Finisher | Female | Intensive |
| AV 50-4 | BVDV | 1:128 | Avellino | Finisher | Male | Intensive |
| AV 53-5 | BVDV | 1:16 | Avellino | Finisher | Female | Intensive |
| AV 59-6 | BVDV | 1:16 | Avellino | Finisher | Female | Intensive |
| A 84-7 | BVDV | 1:8 | Benevento | Finisher | Female | Intensive |
| AV 136-8 | BVDV | Negative | Avellino | Finisher | Female | Intensive |
| PL 43-0 | BVDV | 1:16 | Caserta | Finisher | Male | Intensive |
| PL 43-1 | BVDV | 1:16 | Caserta | Adult | Male | Intensive |
| PL 43-9 | BVDV | 1:16 | Salerno | Finisher | Male | Intensive |
| A 46 | SBV | Negative | Benevento | Growner | Female | Intensive |
| B 6 | SBV | Negative | Benevento | Adult | Female | Intensive |
| B 11 | SBV | 1:256 | Napoli | Adult | Female | Intensive |
| B 14 | SBV | 1:64 | Napoli | Adult | Female | Intensive |
| B 15 | SBV | 1:256 | Napoli | Adult | Female | Intensive |
| B 17 | SBV | 1:256 | Napoli | Adult | Female | Intensive |
| B 18 | SBV | 1:256 | Avellino | Adult | Female | Intensive |
| B 19 | SBV | 1:16 | Salerno | Adult | Female | Intensive |
| B 20 | SBV | 1:32 | Salerno | Adult | Female | Intensive |
| B 32 | SBV | 1:128 | Napoli | Adult | Female | Intensive |
| B 35 | SBV | 1:32 | Napoli | Adult | Female | Intensive |
| B 37 | SBV | Negative | Napoli | Adult | Female | Intensive |
| B 41 | SBV | 1:256 | Napoli | Adult | Female | Intensive |
| AV 56 | SBV | 1:8 | Avellino | Finisher | Male | Intensive |
| B 71 | SBV | 1:32 | Caserta | Finisher | Male | Estensive |
| B 101 | SBV | 1:32 | Salerno | Finisher | Female | Estensive |
| B 102 | SBV | 1:64 | Salerno | Adult | Male | Estensive |
| B 103 | SBV | 1:64 | Salerno | Finisher | Male | Intensive |
| B 105 | SBV/BVDV | 1:32/1:16 | Salerno | Adult | Female | Intensive |
| B 111 | SBV | 1:128 | Salerno | Adult | Male | Intensive |
| PL 01-0 | SBV | 1:16 | Caserta | Finisher | Male | Intensive |
| PL 29-1 | SBV | 1:16 | Caserta | Adult | Female | Intensive |
| AV 64 | Coxiella | Negative | Avellino | Finisher | Female | Intensive |
| AV 71 | Coxiella | 1.69 (Ph II) | Avellino | Finisher | Female | Intensive |
| AV 74 | Coxiella | 0.69 (Ph II) | Avellino | Finisher | Maale | Intensive |
| AV 77 | Coxiella | 0.71 (Ph II) | Avellino | Finisher | Female | Intensive |
| AV 137 | Coxiella | Negative | Avellino | Adult | Male | Intensive |
| PL 14-2 | Coxiella | Negative | Caserta | Finisher | Male | Intensive |
| B 23 | Coxiella | 3.36 (Ph II) | Napoli | Adult | Female | Intensive |
| B 30 | Coxiella | 1.69 (Ph II) | Napoli | Adult | Female | Intensive |
| B 31 | Coxiella | 2.01 (Ph II) | Napoli | Adult | Female | Intensive |
| B 53 | Coxiella | 0.84 (Ph II) | Napoli | Growner | Male | Intensive |
| B 63 | Coxiella | Negative | Napoli | Adult | Male | Intensive |
| 84013-5 | Coxiella | 0.46 (Ph I); 4.25 (Ph II) | Caserta | Finisher | Male | Estensive |
| B 112 | Coxiella | 2.17 (Ph II) | Salerno | Adult | Male | Intensive |
| B 123 | Coxiella | Negative | Caserta | Finisher | Male | Intensive |
| B 125 | Coxiella | 1.19 (Ph II) | Caserta | Finisher | Male | Intensive |
| B 128 | Coxiella | 2.76 (Ph II) | Caserta | Finisher | Male | Intensive |
| B 151 | Coxiella | 0.97 (Ph II) | Caserta | Finisher | Male | Intensive |
|  |  |  |  |  |  |  |
